# Supplementary material for: OpenMM 7: Rapid development of high performance algorithms for molecular dynamics
Source: PLoS Comput Biol. 2017 Jul 26;13(7):e1005659. doi: 10.1371/journal.pcbi.1005659 (PMC5549999; doi:10.1371/journal.pcbi.1005659)
Supplement: S1 Source Code — (ZIP) [file pcbi.1005659.s002.zip › OpenMM-7.0.1-Source/docs/api-python/generated/simtk.openmm.app.gromacsgrofile.GromacsGroFile.html]

GromacsGroFile — OpenMM 7.0.1.dev-5e86c4f documentation


# GromacsGroFile¶

*class* `simtk.openmm.app.gromacsgrofile.``GromacsGroFile`(*file*)¶
:   GromacsGroFile parses a Gromacs .gro file and constructs a set of atom positions from it.

    A .gro file also contains some topological information, such as elements and residue names,
    but not enough to construct a full Topology object. This information is recorded and stored
    in the object’s public fields.

    `__init__`(*file*)¶
    :   Load a .gro file.

        The atom positions can be retrieved by calling getPositions().

        |  |  |
        | --- | --- |
        | Parameters: | **file** (*string*) – the name of the file to load |

    Methods

    |  |  |
    | --- | --- |
    | `__init__`(file) | Load a .gro file. |
    | `getNumFrames`() | Get the number of frames stored in the file. |
    | `getPeriodicBoxVectors`([frame]) | Get the vectors defining the periodic box. |
    | `getPositions`([asNumpy, frame]) | Get the atomic positions. |
    | `getUnitCellDimensions`([frame]) | Get the dimensions of the crystallographic unit cell. |

    `__delattr__`¶
    :   x.\_\_delattr\_\_(‘name’) <==> del x.name

    `__format__`()¶
    :   default object formatter

    `__getattribute__`¶
    :   x.\_\_getattribute\_\_(‘name’) <==> x.name

    `__hash__`¶

    `__reduce__`()¶
    :   helper for pickle

    `__reduce_ex__`()¶
    :   helper for pickle

    `__repr__`¶

    `__setattr__`¶
    :   x.\_\_setattr\_\_(‘name’, value) <==> x.name = value

    `__sizeof__`() → int¶
    :   size of object in memory, in bytes

    `__str__`¶

    `getNumFrames`()¶
    :   Get the number of frames stored in the file.

    `getPositions`(*asNumpy=False*, *frame=0*)¶
    :   Get the atomic positions.

        |  |  |
        | --- | --- |
        | Parameters: | - **asNumpy** (*boolean=False*) – if true, the values are returned as a numpy array instead of a list   of Vec3s - **frame** (*int=0*) – the index of the frame for which to get positions |

    `getPeriodicBoxVectors`(*frame=0*)¶
    :   Get the vectors defining the periodic box.

        |  |  |
        | --- | --- |
        | Parameters: | **frame** (*int=0*) – the index of the frame for which to get the box vectors |

    `getUnitCellDimensions`(*frame=0*)¶
    :   Get the dimensions of the crystallographic unit cell.

        |  |  |
        | --- | --- |
        | Parameters: | **frame** (*int=0*) – the index of the frame for which to get the unit cell dimensions |

High performance molecular simulation on GPUs


### Navigation

- Application Layer
  - Loaders and Setup
  - Representation and Manipulation
  - Simulation
  - Reporting Output
  - Extras
- Library Layer

©2015, Stanford University and the Authors.
|
Powered by Sphinx 1.3.1
& Alabaster 0.7.7
|
Page source
